# Reasons for Exclusion From Intravenous Thrombolysis in Acute Ischemic Stroke: Experience From a Moroccan Stroke Unit

**DOI:** 10.7759/cureus.33248

**Published:** 2023-01-02

**Authors:** Bouchal Siham, Najmi Imane, Benjabara Hanae, Chtaou Naima, Belahsen Faouzi

**Affiliations:** 1 Neurology, Hassan II University Hospital, Fez, MAR

**Keywords:** rapidly improvement stroke, minor stroke, contraindications, intravenous thrombolysis, ct angiography scan, acute ischemic stroke

## Abstract

Background and objective

The rate of intravenous thrombolysis (IVT) in acute ischemic stroke (AIS) is still low due to several absolute and relative contraindications, including admission time delay, which remains the main reason for exclusion from thrombolysis. In this study, we aimed to identify reasons for non-thrombolysis at our stroke center.

Methods

This retrospective study included all patients with a final diagnosis of AIS as per our stroke prospective register from 2014 to 2019. Reasons for non-thrombolysis were analyzed for all AIS and for patients admitted within 4.5 hours from symptom onset. From 2014 to 2016, a non-contrast CT scan was the unique imaging modality used to decide on performing IVT. In 2017, CT angiography was added to the imaging protocol.

Results

Among 3,562 patients with AIS, 3,365 (94.4%) were excluded from thrombolysis; 2,871 (80.6%) were admitted out of the IVT time window, which represents the main reason for exclusion from thrombolysis. Thrombolysis alert (TA) was triggered for 691 (19.4%) patients, and 197 patients had IVT (which represents 28.5% of TA and 5.5% of all AIS). Minor stroke and rapidly improving symptoms of stroke were also reasons for non-thrombolysis, which explain the high-average initial National Institutes of Health Stoke Scale (NIHSS) score of more than 12 in the thrombolysis group. CT angiography allows for the analysis of the supra-aortic trunks, the circle of Willis, and the collateral status. Therefore, during the period when a CT angiography scan was used, there were more IVTs for minor strokes, rapidly improving strokes, and AIS patients admitted beyond the IVT time window.

Conclusions

This study highlights the common reasons for exclusion from thrombolysis. Efforts should be undertaken to avoid admission time delays. Also, based on our findings, minor stroke and improving stroke no longer represent absolute contraindications for IVT in AIS.

## Introduction

Acute ischemic stroke (AIS) is a major cause of neurological disability. Fast reperfusion, including intravenous thrombolysis (IVT) and mechanical thrombectomy, remain the most effective therapeutic methods in standard care. However, many early-admitted patients do not benefit from IVT due to several reasons.

One of the main reasons for exclusion from thrombolysis is admission time delay [1-2]. The permissible time delay for IVT has been extended from three hours to 4.5 hours after symptom onset. The randomized, placebo-controlled European Cooperative Acute Stroke Study III (ECASS III) has proven that patients treated with intravenous recombinant tissue plasminogen activator (rt-PA) up to 4.5 hours had a significantly better outcome at day 90 than controls, without increased mortality rate and no excess risk of symptomatic intracranial hemorrhage [3-4].

Some absolute contraindications have become relative over time, which has led to an increase in the rate of IVT in AIS, such as advanced age, rapidly improving or mild stroke (RIMS), seizures, and recent surgery [1]. However, the rate of IVT remains low because of several factors apart from these exclusion criteria, such as the lack of public awareness and stroke centers [5]. In light of this, we aimed to identify the reasons for non-thrombolysis in AIS in Moroccan stroke centers.

## Materials and methods

Study assessment criteria

All patients who presented with a final diagnosis of AIS to the emergency or the neurology departments of the Hassan II University Hospital, Fez, Morocco, from December 12, 2014, to June 11, 2019, were retrospectively included in the study using our stroke prospective register. Patients underwent anamnestic, general evaluation (blood pressure and sugar), and neurological evaluation was performed by the emergency physicians, and then by the neurologist. Stroke severity was evaluated by the National Institutes of Health Stroke Scale (NIHSS) score. A minor stroke was defined as a score ≤6.

For patients admitted within 4.5 hours from stroke onset, a thrombolysis alert (TA) was triggered. Brain imaging with a non-contrast CT scan was performed in all patients to confirm AIS; MRI was also used in some patients. The radiologist and the neurologist evaluated early ischemic signs using the Alberta Stroke Program Early CT Score (ASPECTS) [6]. A cutoff score ≤7 suggests the involvement of one-third of the middle cerebral artery territory by the infarct. For patients with a “wake up” stroke or unknown delay from stroke onset, a brain MRI was performed, including diffusion-weighted imaging (DWI), fluid-attenuated inversion recovery (FLAIR) images, time of flight images, and T2-weighted images. 

In 2017, we started using CT angiography to study the supra-aortic trunks, the circle of Willis, and collaterals quality. CT angiography is done systematically and immediately after a cerebral CT scan and before rt-PA administration. Concurrent CT and CT angiography does not significantly delay the time to IVT (three minutes more) and helps us make decisions regarding the site of occlusion and collateral status. CT angiography can also reveal some rare contraindications of IVT such as aortic arch dissection and free-floating thrombus of TSA.

The patients underwent biological examinations including the mandatory international normalized ratio (INR) tests if they were treated by anticoagulants (vitamin K antagonists). The neurologist evaluated the inclusion and exclusion criteria for IVT. All patients or their family members provided written informed consent before the administration of thrombolytic drugs.

We calculated the total number of AIS received during the study period, triggered TAs, and performed IVTs. For all AIS, we analyzed the causes for not giving thrombolysis. For TA, the following baseline characteristics were recorded from our prospective alerts register: age, sex, initial NIHSS score, type of brain imaging, onset-to-door time (ODT), and door-to-imaging time (DIT).

The use of the CT angiography scan in TA allowed us to divide this series into two periods [from December 12, 2014, to May 31, 2017 (without a CT angiography scan); and from June 1, 2017, to June 11, 2019 (with a CT angiography scan)], in order to compare the reasons for exclusion from thrombolysis in each period. Since 2017, annual campaigns have been organized among the Moroccan population to raise awareness of stroke signs and the benefits of timely access to emergency medical care.

Thrombolysis alert procedure at our hospital

The service of pre-hospital medical transport is not yet available in our country, and patients arrive from home by their own means of transport (car, taxi, or private ambulance). The TA is triggered once the patients arrive at the hospital. Once the patient is at the hospital door, an emergency physician has to determine if the situation is urgent. If the patient is suspected of having a stroke within a therapeutic time window of IVT, that patient is admitted to the emergency room and the neurologist is called immediately. The entire team (emergency physician, neurologist, and radiologist) is available 24/7. The emergency physician assesses the patient's vital functions (blood pressure, glycemia, pulse rate, respiration), employs the peripheral intravenous catheter, and performs EKG. The neurologist obtains the medical history and examines the patient and he calls the radiologist to make the CT scan available as soon as possible. There is only one CT scan for all emergency situations. After obtaining a CT scan and CT angiography, the decision to administer rt-PA is made by the neurologist, and the patient is admitted to the stroke unit.

Ethical consideration

The Ethical Committee of the Hassan II University Hospital approved the study.

## Results

During the five-year study period, 3,562 patients had a final diagnosis of ischemic stroke (IS) of which 3,365 (94.4%) did not receive thrombolysis; 2,871 (80.6%) of these patients were admitted out of the thrombolysis time window, which represents the main reason for exclusion from thrombolysis in our series. The other common reasons were large infarct on imaging (ASPECTS ≤7) (6.7%), minor stroke (2.9%), intra-hospital delay (1.7%), and high bleeding risk (INR >1.7 or prior use of heparin) (0.6%). All reasons for exclusion from thrombolysis in all IS are presented in Table 1.

**Table 1:** Reasons for exclusion from thrombolysis in acute ischemic stroke (n=3,562) ASPECTS: Alberta Stroke Programme Early CT Score; NIHSS: National Institutes of Health Stoke Scale; INR: international normalized ratio

Reasons for exclusion	N (%)
The prehospital thrombolysis time window exceeded	2,871 (80.6%)
Large infarct on imaging (ASPECTS cutoff of ≤7)	262 (6.7%)
Minor deficit (NIHSS score ≤6)	104 (2.9%)
Intra-hospital thrombolysis time window exceeded (>4.5 hours)	63 (1.7%)
Absence of cerebral artery occlusion on imaging	37 (1%)
High bleeding risk (INR >1.7 or prior use of heparin)	24 (0.6%)
Rapidly improving symptoms	9 (0.2%)
Recent myocardial infarction (<3 weeks)	5 (0.1%)
Recent surgery (<14 days)	4 (0.1%)
Dissection	3 (0.08%)
Family refusal	2 (0.05%)
Lacunar infarct	1 (0.02%)
High blood pressure (systolic blood pressure ≥185 mmHg or diastolic blood pressure ≤110 mmHg)	1 (0.02%)

TA was triggered for 691 patients (19.4%) with a diagnosis of AIS; 197 patients benefited from IVT, which represents 28.5% of TA and 5.5% of all AIS.

**Figure 1:**
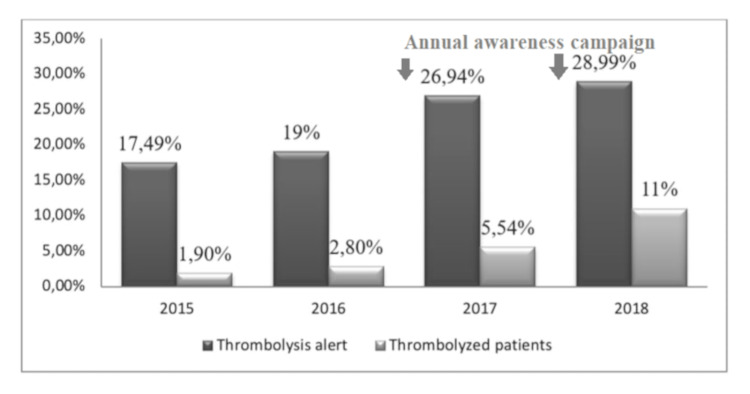
Rates of thrombolysis alerts and thrombolyzed patients over the years

The annual rate of TA increased from 17.4% in 2015 to 28.9% in 2018. The rate of thrombolysis also increased from 1.9% in 2015 to 11% in 2018. The awareness campaigns started in 2017 seem to have played a role in this increase in TA numbers (Figure 1).

For the two groups of thrombolyzed and non-thrombolyzed patients with AIS for whom a TA was triggered, the mean age was 67.2 ± 12.2 years (range: 19-90 years) and 65.2 ± 13.9 years (range: 21-110 years) respectively. There was no statistically significant difference between the two groups in terms of age and sex (p>1). The mean NIHSS score was 13.5 ± 4.3 in the group of thrombolyzed patients versus 10.72 ± 6.1 in the group of non-thrombolyzed patients. The severity of stroke was statistically associated with thrombolysis. An initial NIHSS score of >12 was found more frequently in thrombolyzed patients than in non-thrombolyzed patients (60.2% and 44.2% respectively, p=0.0001). The mean ODT was 122.6 ± 71.3 minutes for patients receiving IVT and 150.7 ± 88.4 minutes for non-thrombolyzed patients. The mean DIT was 22.9 ± 21.3 minutes for thrombolyzed patients and 23.4 ± 22.2 minutes for non-thrombolyzed patients. There was no statistically significant difference between the two groups in terms of time delay. CT angiography was performed in 55.3% of thrombolyzed patients and 48.9% of non-thrombolyzed patients. The baseline characteristics of patients for whom a TA was triggered are listed in Table 2.

**Table 2:** Baseline characteristics of patients for whom a thrombolysis alert was triggered SD: standard deviation; ODT: onset-to-door time; NIHSS: National Institutes of Health Stroke Scale; DIT: door-to-imaging time; CT: computed tomography; MRI: magnetic resonance imaging

Variables	Thrombolyzed patients (n=197)	Non-thrombolyzed patients (n=494)
Age (years)		
Mean ± SD	67.2 ± 12.2	65.2 ± 13.9
Range	19-90	21-110
Sex		
Male, n (%)	108 (55.1%)	219 (44.2%)
Female, n (%)	89 (44.4%)	275 (55.6%)
Sex ratio	1.24	1.25
ODT (minutes)		
Mean ± SD	122.6 ± 71.3	150.7 ± 88.4
Range	0-570	0-485
≤270, n (%)	194 (98.4%)	468 (94.7%)
>270, n (%)	3 (1.52%)	26 (5.2%)
NIHSS score		
Mean ± SD	13.5 ± 4.3	10.7 ± 6.1
Range	2-23	0-23
≤6, n (%)	15 (7.7%)	148 (29.9%)
7-12, n (%)	63 (31.6%)	127 (25.7%)
>12, n (%)	119 (60.2%)	219 (44.2%)
DIT (minutes)		
Mean ± SD	22.98 ± 21.3	23.4 ± 22.2
Range	0-165	0-190
Brain imaging		
CT scan, n (%)	86 (43.6%)	248 (50.2%)
CT angiography scan, n (%)	109 (55.3%)	242 (48.9%)
MRI, n (%)	1 (0.5%)	3 (0.6%)

The analysis of the TA series in the first period when we used a non-contrast CT scan and in the second period when we started using a CT angiography scan revealed that the rates of TA and IVT increased from 43.2% to 56.8% and from 15.5% to 25.47% respectively. While there were 211 non-thrombolyzed patients in the first period, the second period had 283 patients. The main reasons for exclusion from thrombolysis were similar in the two groups, including acute large infarct on imaging (49.2% and 48.3% respectively), minor or rapidly improving symptoms (30% and 17.3% respectively), intra-hospital thrombolysis time window exceeded (11.8% and 13.4% respectively), and high bleeding risk (4.2% and 5.3% respectively).

Patients with minor and rapidly improving symptoms started receiving IVT more frequently after we started using CT angiography (30.3% versus 17.3%, p=0.001). Thirteen patients were thrombolyzed outside the therapeutic time window, and the majority of them (11/13) were thrombolyzed in the second period, and this difference was statistically significant (p=0.008). The comparison of reasons for non-thrombolysis in AIS between the first and the second period of the study is presented in Table 3.

**Table 3:** Reasons for exclusion from non-thrombolysis in AIS in the first and the second periods of the study AIS: acute ischemic stroke; ASPECTS: Alberta Stroke Programme Early CT Score; NIHSS: National Institutes of Health Stoke Scale; INR: international normalized ratio

Variables	First period, n (%)	Second period, n (%)	P-value
Thrombolysis alert in ischemic stroke (n=691)	274 (39.6%)	417 (60.3%)	-
Thrombolyzed patients (n=197)	63 (31.9%)	134 (68%)	0.009
Reasons for exclusion from thrombolysis			
Acute large imaging (ASPECTS cutoff of ≤7)	104 (49, 2%)	137 (48.3%)	1
Minor or improving deficit (NIHSS score ≤6)	64 (30%)	49 (17.3%)	0.001
Intra-hospital thrombolysis time window exceeded (>4.5 hours)	25 (11.8%)	38 (13.4%)	0.12
High bleeding risk (INR >1.7 or prior use of heparin)	9 (4.2%)	15 (5.3%)	1
Recent myocardial infarction (<3 weeks)	3 (1.4%)	2 (0.7%)	1
Recent surgery (<14 days)	3 (1.4%)	1 (0.3%)	1
Family refusal	2 (0.9%)	0 (0%)	1
Lacunar infarct	0 (0%)	1 (0.3%)	1
High blood pressure (systolic blood pressure ≥185 mmHg or diastolic blood pressure ≤110 mmHg)	1 (0.4%)	0 (0%)	1

## Discussion

Our analysis revealed that time delay in admission is the main reason for exclusion from thrombolysis, which aligns with the findings in the literature [1,2,6]. This time delay could be significantly improved by raising public awareness, better organization of medical transport, and increasing the number of stroke centers. Continuous medical education on stroke for general practitioners (GPs) and neurologists could also increase the number of thrombolysis for patients. Since 2017, our center has been organizing annual awareness campaigns on the occasion of World Stroke Day, which has contributed to an increase in the annual rate of thrombolysis. These campaigns have confirmed their effectiveness in raising the knowledge related to warning symptoms of stroke and the importance of quick emergency consultation, consequently increasing the number of thrombolyzed patients [7].

With respect to TA, our analysis of the first period when we used only non-contrast CT scans for decision making and the second period when we started using CT angiography scans revealed similar reasons for exclusion from thrombolysis; but there was an increase in the rate of IVT.

Time delay is no longer an absolute contraindication for IVT and has become a relative one. This has been confirmed by the findings of the EXTEND (Extending the Time for Thrombolysis in Emergency Neurological Deficits) clinical trial, which employed CT perfusion scanning for identifying hypoperfused but salvageable regions of the brain, which enabled extending the time window until nine hours [8].

Another method to evaluate salvageable parenchyma without a CT perfusion scan is to use CT angiography for studying and scoring collateral status. Leptomeningeal collaterals have been considered as having a beneficial role in patients with AIS despite various scoring scales and different imaging modalities [9]. The collaterals are an important determinant of tissue outcome [10].

In a study investigating how time affects penumbral salvage and infarct growth in untreated acute IS patients and whether collateral status affects this relationship, it was found that collateral flow strongly dictates the natural evolution of ischemic injury irrespective of the timing of assessment of the penumbra. Better collaterals were associated with larger penumbral salvage and decreased infarct growth in untreated stroke patients within 24 hours of stroke onset. However, time from stroke onset was neither associated with penumbral salvage nor infarct growth. The natural evolution of penumbra is modified by the complex interplay of time, vessel occlusion, recanalization status, and collateral flow. These findings further support the use of IVT and endovascular treatment in extended time windows. It seems that the collateral clock is more important than the time clock for tissue fate [11].

The use of CT angiography, which is noninvasive and widely available in emergency situations, to evaluate collaterals status helps in selecting eligible patients for treatment in AIS in stroke centers without perfusion software [12]. At our center, scoring collateral status allowed us to give rt-PA for patients up to 4.5 hours and increase the total number of thrombolyzed patients. RIMS was the most common reason that patients, arriving within the three-hour time window, are excluded from IVT [13-15].

Previous studies have demonstrated that a substantial proportion of patients excluded from treatment with rt-PA because of RIMS have poor neurologic and functional outcomes at three months [14,16]. Persisting large-vessel occlusion is a major predictor of early neurologic deterioration with infarct expansion and poor functional outcome at discharge in patients with RIMS because of collateral flow failure and hemodynamic compromise [13]. Many studies have shown the safety and efficacy of intravenous rt-PA in RIMS. Most patients treated with rt-PA achieved good outcomes, with some recovering without any persisting symptoms [17-20]. The risk of symptomatic intracerebral hemorrhage after thrombolysis in patients with mild stroke or rapidly improving symptoms was relatively low, which suggests that the benefit of IVT outweighs the risk in patients with RIMS [20,21]. RIMS with vessel occlusion should not be excluded from thrombolysis because of the risk of deterioration.

Our study has a few limitations. Primarily, this was a retrospective study of prospectively acquired data. Moreover, our data were drawn from a single center.

## Conclusions

Admission time delay remains the main reason for exclusion from thrombolysis in AIS. In our setting, raising public awareness, better organization of medical transport, and increasing the number of stroke centers could reduce the reasons for non-thrombolysis. Relative contraindications should not exclude some patients who are admitted within the time window from thrombolysis, in order to raise the rate of IVT in AIS. The use of CT angiography could expand the therapeutic time window for patients without other contraindications for thrombolysis.
